# Adherence status to Adjuvant Endocrine Therapy in Chinese Women with Early Breast Cancer and its influencing factors: A cross‐sectional survey

**DOI:** 10.1002/cam4.3017

**Published:** 2020-04-01

**Authors:** Hui Xu, Feng Jin, Xiu‐jie Zhang, Da‐qiu Wang, Shao‐fen Yu, Ai‐ping Wang

**Affiliations:** ^1^ Cancer hospital of China Medical University Liaoning Cancer Hospital & Insititute Shenyang China; ^2^ The First Affiliated Hospital of China Medical University Shenyang China; ^3^ The First affiliated Hospital of Dalian Medical University Dalian China; ^4^ Liaoning University of Traditional Chinese Medicine Shenyang China

**Keywords:** adherence, breast cancer, compliance, endocrine therapy, oncology, persistence

## Abstract

**Objective:**

Despite the proven benefits of adjuvant endocrine therapy, adherence to oral endocrine therapy in breast cancer treatment is a substantial problem. The aim of this study was to assess adherence to adjuvant endocrine therapy by women in China for the first 5 years, and to identify its influencing factors.

**Methods:**

Stratified sampling method was adopted to select 1875 cases of breast cancer patients for cross‐sectional telephone follow‐up. Compliance to medications was assessed using the Morisky Medication Adherence Scale. Status of endocrine therapy was assessed using nine additional questions. Binomial regression was used when assessing the factors associated with persistence, multinomial regression models were used to assess factors associated with compliance.

**Results:**

Of 888 patients who started adjuvant endocrine therapy, 769(86.6%) persisted and 119 (13.4%) discontinued. 760 patients who completed Morisky Medication Adherence Scale, the compliance was 7.4% low, 42% medium, and 50.6% high. The type of medication, duration of medication and side effects had an impact both on persistence and compliance. Age, history of radiotherapy and caregivers only had an impact on persistence.

**Conclusions:**

Medication adherence was affected by many factors. Special attention and interventions should be given to women taking tamoxifen in the 2nd to 3rd year of medication, and aromatase inhibitors in the 1st to 2nd year. Further prospective design studies are needed to explore effective measures to improve medication adherence of women with breast cancer treated by endocrine therapy.

## Graphical abstract

Of 888 patients who started adjuvant endocrine therapy, 769(86.6%) persisted and 119 (13.4%) discontinued. 760 patients who completed MMAS, the compliance was 7.4% low, 42% medium, and 50.6% high. Adherence was affected by many factors. Special attention and interventions should be given to women taking tamoxifen in the 2nd to 3rd year of medication, and AIs in the 1st to 2nd year.

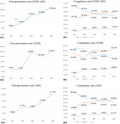

## 1 BACKGROUND

Breast cancer (BrCa) is the most common cancer among women worldwide.^1^, ^2^ GLOBOCAN 2018 showed that there would be about 2.1 million newly diagnosed female breast cancer cases in 2018, accounting for almost one in four cancer cases among women. The disease is the most frequently diagnosed cancer in the vast majority of the countries (83.24%) and is also the leading cause of cancer‐related death in over 100 countries.^3^ Approximately two‐thirds of breast cancer patients test positive for the estrogen receptor (ER) and/or progesterone receptor (PR).^4^ For this majority of patients, adjuvant endocrine therapy (AET) such as tamoxifen (TAM) and aromatase inhibitors (AIs) have proven clinical benefit. AET can significantly reduce recurrence and mortality in BrCa women who are hormone receptor‐positive.^5^, ^6^, ^7^ Updated clinical practice guidelines recommend extending AET use from 5 to 10 years.^4^, ^8^ Despite the radical difference made by AET in BrCa outcomes, up to 50% of women do not adhere to prescribed regimens^9^, ^10^ and 31%‐73% of women are nonpersistent with AET.^11^, ^12^, ^13^ Taking < 80% of AET doses has been associated with a 20% increased mortality risk.^14^ Adherence to oral endocrine therapy in BrCa treatment is a substantial problem.^15^ Therefore, identifying possible reasons for nonpersistence and nonadherence in women with BrCa is vital.

The World Health Organization (WHO) defines adherence as “the extent to which a person's behavior—taking medication, following a diet, and/or executing lifestyle changes, corresponds with agreed recommendations from a health care provider”.^16^

Medication adherence is defined as a composite of compliance (how well physician's orders are followed) and persistence (how long an individual continues on prescribed therapy).^17^

The aim of this study was to assess adherence to AET by women in China for the first five years and to identify its influencing factors, so as to provide improvement measures to ensure the efficacy of endocrine therapy in the future.

## 2 METHODS

### 2.1 Participants

A total of 9128 cases of BrCa were admitted from January 2013 to December 2017 from a university cancer hospital in Shenyang, China. Stratified random sampling method was adopted to select 375 cases of breast cancer patients each year; a total of 1875 cases were screened to meet the following inclusion and exclusion criteria.

Inclusion criteria included positive for the estrogen receptor (ER) and/or progesterone receptor (PR); newly diagnosed with breast cancer; receiving oral endocrine therapy drugs; women above 18 years old; and volunteered participation in the survey. Exclusion criteria included patients with a history of other malignant tumors or patients with distant metastasis.

### 2.2 Measures

*Demographic & clinical variables* were obtained through clinical record review: the tumor characteristics noted included stage and previous treatments; patient information at diagnosis included common epidemiological characteristics (age, height, weight, ethnicity, marital status, and medical insurance.) and Charlson morbidity score. Self‐reported demographic data (employment status, occupation, smoking, drinking, and medical payment methods.) were also included. Body mass index (BMI) and Charlson morbidity score^18^ at diagnosis were calculated for each woman.

*Compliance to medications* was assessed using the four‐item Morisky Medication Adherence Scale (MMAS);^19^ the MMAS showed good psychometric properties (Cronbach's alpha of 0.61) in the original validation study. The answer of the questionnaire include "yes" and "no". When the answer is "yes", the score is 0, and "no" is 1. The higher the score, the worse the compliance, with 0 indicating high compliance, 1‐2 indicating medium compliance, and 3‐4 indicating low compliance. Chinese researchers translated and revised MMAS into Chinese version, and we tested the internal consistency before use, Cronbach's alpha of the modified questionnaire was 0.724.

*Status* and *persistence of endocrine therapy* assessed using nine additional questions designed by the authors included: Are you still taking endocrine therapy medications? When did you begin endocrine therapy? What's the name of your endocrine medication? whether switched the medicine? Have you ever missed to take any medicine? If so, why and when? How many days (or times) do you miss medication per month? (no missed dose, 1‐5 missed doses, and more than 6 missed doses) Do you have any discomfort with this medicine? Do you know the side effects of endocrine medicine? Do you need others' care?

*Nonpersistence*: Women were considered discontinuers if they reported they were no longer using TAM or AIs and their self‐reported duration of use was <5 years after breast cancer diagnosis. Women who reported 5 or more years of TAM and/or AIs use (ie, completers), or who reported current TAM or AIs use (ie, continuers) no matter whether or not they switched medication at the time of the survey (even if they reported <5 years of use) were classified as women who did not discontinue.

### 2.3 Data collection

This was a cross‐sectional, observational study conducted between July 2018 and September 2018. Our study was approved by the ethics committee at Liaoning Cancer Hospital. The author (Xu Hui) conducted a telephone follow‐up demonstration before the start of follow‐up, and unified instructions and a list of follow‐up procedures. Two graduate students (Xiujie Zhang and Daqiu Wang) who had follow‐up experience received training regarding the survey instrument and data collection methods. They completed the telephone follow‐up after passing the training. Participants had adequate information regarding the purpose of the research. They had the right of free choice, enabling them to voluntarily consent or decline participation in the research. Confidentiality was maintained. Eligible participants completed a telephone follow‐up survey on factors associated with endocrine therapy. Each contact number was dialed at different times on different dates for three consecutive times. If no connection could be made for three times, the telephone follow‐up was considered as a failure.

### 2.4 Statistical analysis

Data were analyzed using IBM SPSS Statistics (version 23). Statistical description includes frequency, percentage, P_50_ (P_25_‐P_75_), etc. Mann‐Whitney U test was used for comparison between the two groups. Kruskal‐Wallis H test (Nemenyi test) was used for comparison of multiple groups, and Nemenyi test was used for further pairwise comparison. *χ* ^2^ test or Fisher's exact test was used for comparison of quantitative data. Binomial regression was used when assessing the factors associated with persistence, multinomial regression models were used to assess factors associated with compliance. First, univariate analysis of variables that may affect patients' adherence with endocrine therapy was conducted. The variables with significant differences in univariate analysis were further analyzed by multivariate Logistic regression analysis to explore the influencing factors of patient compliance. Odds ratios (OR) and 95% confidence intervals (CI) were calculated for each characteristic.

## 3 RESULTS

### 3.1 Sample characteristics

**Figure 1:**
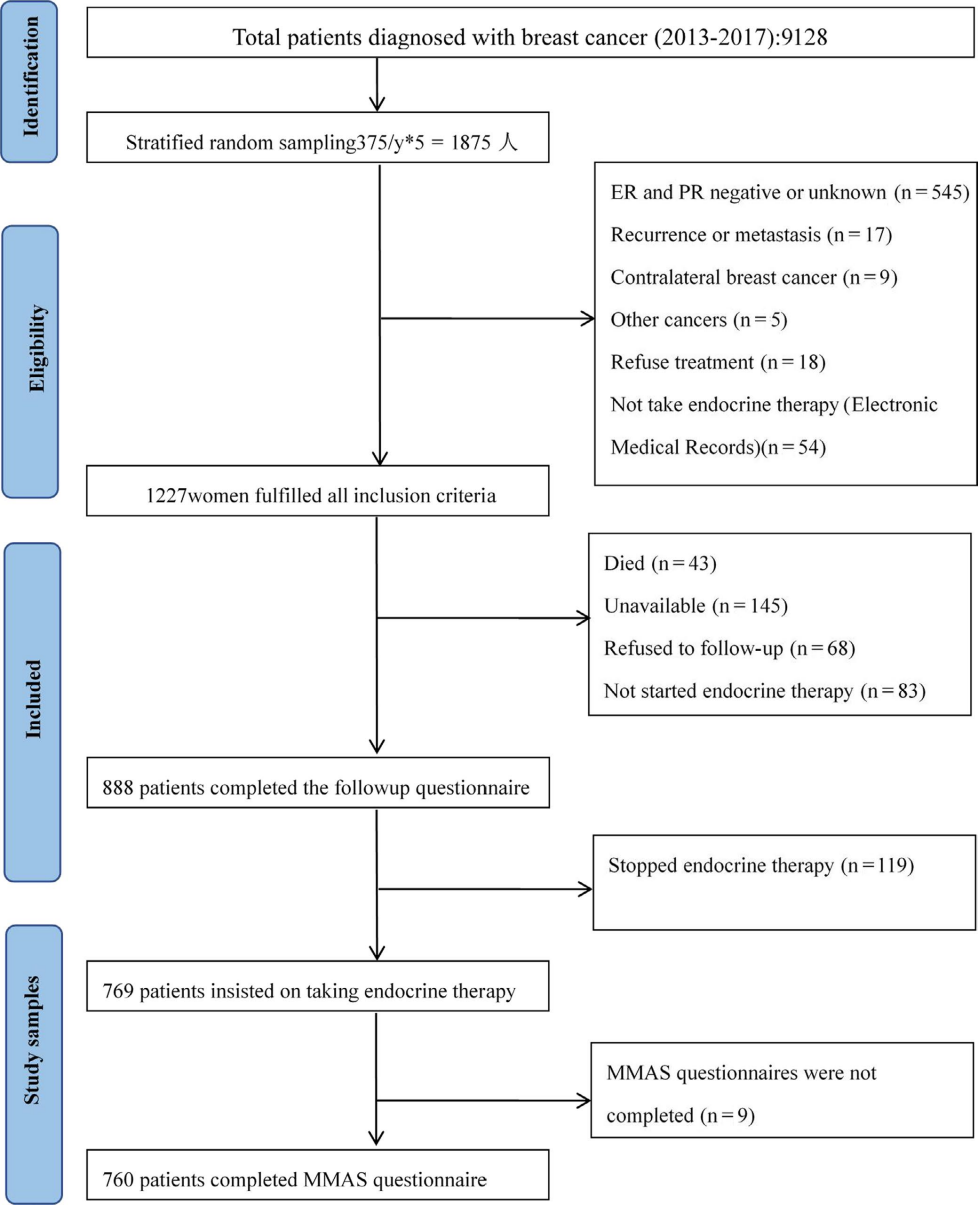
Flowchart of study sample selection

A total of 1227 patients with hormone receptor‐positive breast cancer met the patient selection criteria. Of those, 339 patients were not included in the analysis due to the following reasons: died (N = 43), lost to follow‐up (N = 145), refused to follow‐up (N = 68) and did not take or begin endocrine therapy (N = 83). Thus, we report results for 888 patients; the patient selection criteria are shown in Figure 1. The median age of all patients aged was 54.52 years (range, 27‐89 years), 357 patients took TAM and 531 patients took AIs, the characteristics are shown in Table 1.

**Table 1:** Patient demographics and tumor characteristics

Characteristics	N	%
Age at diagnosis (y)		
Median (interquartile range)	54	47‐62
≤39	57	6.4
40‐49	249	28
50‐59	290	32.7
≥60	292	32.9
Marital status		
Married/partner	878	98.9
Single/divorced	10	1.1
Ethnicity		
Han	823	92.7
Minorities	65	7.3
Smoker		
Never	876	98.6
Current	12	1.4
Drinker		
Never	846	95.3
Current	42	4.7
Employment		
Yes	311	35
No	531	59.8
On work disability	46	5.2
Occupation		
Retiree	277	31.2
Worker	90	10.1
Farmer	143	16.1
Office staff	84	9.5
Professionals	83	9.3
No	119	13.4
Others	92	10.4
Medical insurance		
UB insurance	310	34.9
RC insurance	257	28.9
No insurance	159	17.9
Others	162	18.2
Medication type		
AIs	531	59.8
TAM	357	40.2
Understanding side effects^b^		
Yes	589	66.3
No	202	22.7
Caretakers		
No	426	48
Yes	462	52
BMI (kg/m^2^)		
Mean (SD)	24.8	10.1
<18.5	21	2.4
18.5‐24.99	498	56.1
25‐29.99	316	35.6
≥30	53	6
Radiotherapy		
Yes	302	34
No	586	66
Chemotherapy		
Yes	745	83.9
No	143	16.1
HER2/neu		
Negative	785	88.4
Positive	103	11.6
K167		
<30%	524	59
≥30%	364	41
Charlson morbidity score		
0	700	78.8
1	143	16.1
≥2	45	5.1
pT		
1	258	29.1
2	549	61.8
3	29	3.3
4	18	2
Unknown	34	3.8
pN		
Positive	363	40.9
Negative	499	56.2
No axillary surgery	26	2.9
Duration of medication(M)		
1‐12	205	23.1
13‐24	209	23.5
25‐36	160	18
37‐48	168	18.9
≥49	146	16.4
No. of side effects		
0	412	46.4
1	315	35.5
≥2	140	15.8
Unknown	21	2.4

Abbreviations: RC insurance, Rural Cooperative Medical Insurance; UB insurance, Urban Residents Basic Medical Insurance.

a Fisher's exact probability method was used.

b The variable has a missing value.

### 3.2 Persistence with endocrine therapy

**Figure 2:**
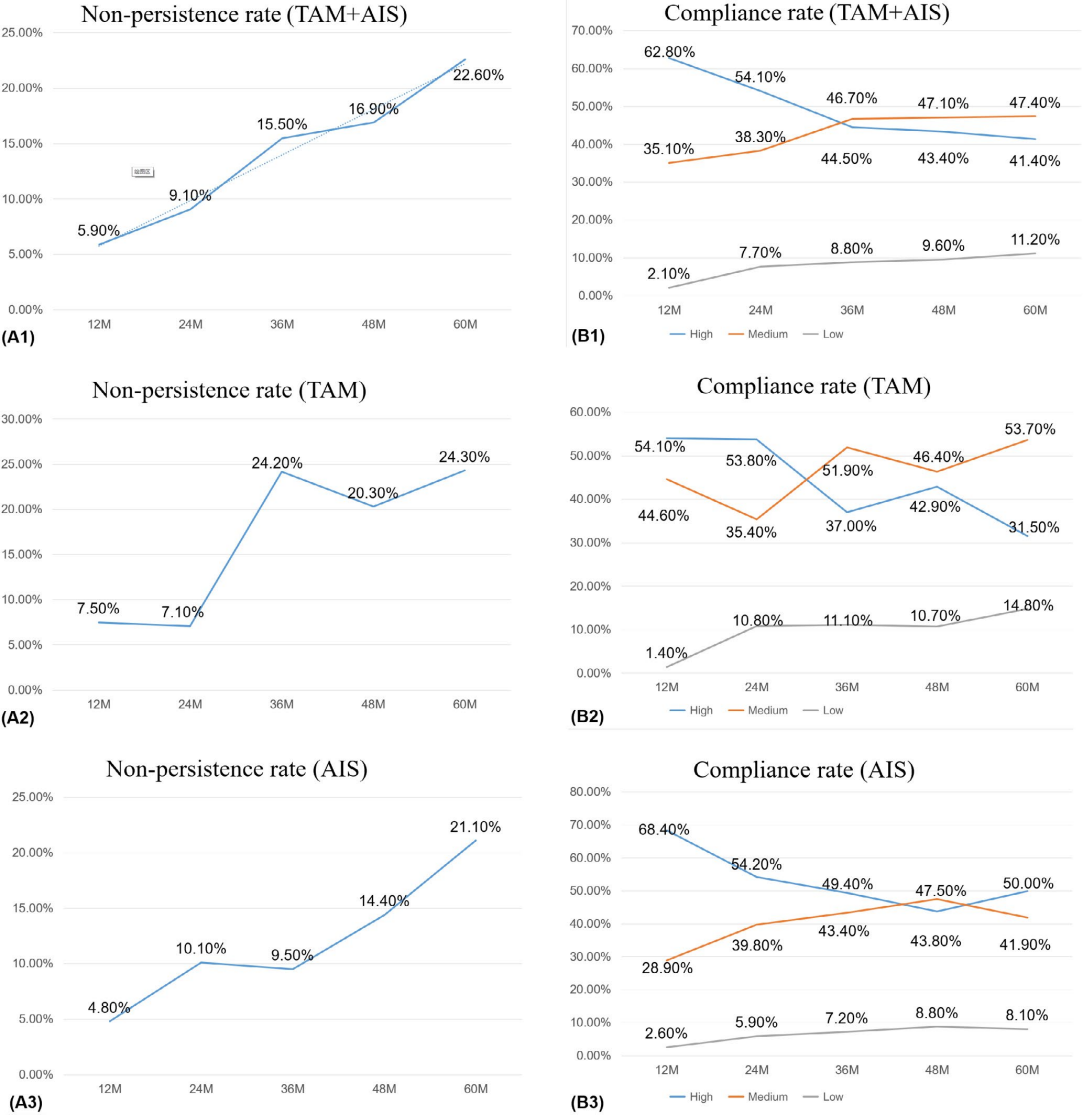
Nonpersistence rate and compliance rate curve

Of 888 patients who started adjuvant endocrine therapy, 769 patients (86.6%) were classified as persistent, 119 patients (13.4%) were considered nonpersistent. The non‐persistence rate from the first year to the fifth year was 5.9%, 9.1%, 15.5%, 16.9% and 22.6%, respectively. Patients taking TAM for 2‐3 years showed obviously non‐persistence, while the non‐persistence rate of the AIs group was obviously increased at the 1‐2 year, the results are shown in Figure 2.

### 3.3 Compliance with endocrine therapy

Of the 769 patients who insisted on taking the medicine, 422 (54.88%) reported consistently taking their medication, with no missed doses, patients who missed less than five doses per month were 263 (34.2%). MMAS assessed compliance among 760 patients who completed the questionnaire, the distribution of compliance for these women was 7.4% low, 42% medium, and 50.7% high. The rate of high compliance ranged from 41.4% to 62.8% at 1 to 5 years, and the rate of low compliance ranged from 2.1% to 11.2%. Compliance fluctuated at 2 to 3 years, the results are shown in Figure 2.

### 3.4 Characteristics associated with persistence and compliance

The analysis showed that the type of medication, duration of medication and side effects had an impact both on persistence and compliance to endocrine therapy for breast cancer. Age, history of radiotherapy, and presence of caregivers only had an impact on persistence to medication. BMI as a continuous variable had an effect on medication compliance, but as ranked data, the effect on medication compliance was not statistically significant. The multivariate analysis of influencing factors results is shown in Tables 2 and 3. Univariate analysis of influencing factors is shown in Additional file 1 and Additional file 2.

**Table 2:** Analysis of influencing factors of medication persistence

Characteristics	Persistence (N = 769)	Nonpersistence (N = 119)	Unadjusted		Adjusted	
			OR (95%CI)	*P*‐value	OR (95%CI)	*P*‐value
Age at diagnosis (y)						
≤39	53 (6.9)	4 (3.4)	1.00	.027	1.00	<.001
40‐49	219 (28.5)	30 (25.2)	1.82 (0.61‐5.38)	.282	1.52 (0.50‐4.61)	.464
50‐59	258 (33.6)	32 (26.9)	1.64 (0.056‐4.84)	.368	1.86 (0.61‐5.66)	.275
≥60	239 (31.1)	53 (44.4)	2.94(1.02‐8.47)	.046	4.83 (1.54‐15.18)	.007
Radiotherapy						
Yes	271 (35.2)	31 (26.1)	1.00			
No	498 (64.8)	88 (73.9)	1.55 (1.00‐2.39)	.050		
Duration of medication(M)						
1‐12	192 (25.0)	13 (10.9)	1.00	<.001	1.00	.002
13‐24	190 (24.7)	19 (16.0)	1.48 (0.71‐3.08)	.297	1.51 (0.71‐3.17)	.283
25‐36	135 (17.6)	25 (21.0)	2.74 (1.35‐5.54)	.005	2.58 (1.26‐5.29)	.010
37‐48	139 (18.1)	29 (24.4)	3.08 (1.55‐6.14)	.001	2.86 (1.42‐5.79)	.003
≥49	113 (14.7)	33 (27.7)	4.31 (2.18‐8.54)	<.001	3.60 (1.79‐7.21)	<.001
Medication type						
AIs	472 (61.4)	59 (49.6)	1.00		1.00	
TAM	297 (38.6)	60 (50.4)	1.62 (1.10‐2.38)	.015	2.91 (1.74‐4.85)	<.001
No. of side effects						
0	366 (47.6)	46 (38.7)	1.00	.028	1.00	.026
1	267 (34.7)	48 (40.3)	1.43(0.93‐2.21)	.106	1.45 (0.92‐2.27)	.107
≥2	122 (15.9)	18 (15.1)	1.17 (0.66‐2.10)	.589	1.29 (0.71‐2.37)	.406
Unknown	14 (1.8)	7 (5.9)	3.98 (1.53‐10.37)	.005	4.37 (1.60‐11.91)	.004
Caretakers						
No	349 (45.4)	77 (64.7)	1.00			
Yes	420 (54.6)	42 (35.3)	0.45 (0.30‐0.68)	<.001		

**Table 3:** Analysis of influencing factors of medication compliance

	Characteristics	Control group N (%)	Comparison group N (%)	Unadjusted		Adjusted	
				OR (95%CI）	*P*‐value	OR (95%CI)	*P*‐value
Medium compliance	Duration of medication(M)						
	1 −12	118 (30.6)	65 (20.4)	1.00		1.00	
	13‐24	100 (26.0)	70 (21.9)	1.27 (0.83‐1.95)	.275	1.28 (0.83‐1.97)	.272
	25‐36	60 (15.6)	64 (20.1)	1.94 (1.22‐3.08)	.005	1.91 (1.19‐3.06)	.007
	37‐48	59 (15.3)	65 (20.4)	2.00 (1.26‐3.18)	.003	1.96 (1.22‐3.14)	.005
	≥49	48 (12.5)	55 (17.2)	2.08 (1.27‐3.40)	.003	2.01 (1.22‐3.3)	.006
	Medication type						
	AIs	249 (64.7)	180 (56.4)	1.00		1.00	
	TAM	136 (35.3)	139 (43.6)	1.41 (1.04‐1.92)	.026	1.41 (1.03‐1.92)	.034
	No. of side effects						
	0	202 (52.5)	133 (41.7)	1.00		1.00	
	1	127 (33.0)	131 (41.1)	1.54 (1.11‐2.14)	.007	1.57 (1.12‐2.19)	.008
	≥2	55 (14.3)	54 (16.9)	1.25 (0.81‐1.95)	.072	1.24 (0.8‐1.95)	.337
	Unknown	1 (0.3)	1 (0.3)	5.94 (0.66‐53.73)	.768	4.14 (0.45‐38.2)	.211
	BMI	385 (100.0)	319 (100.0)	0.96 (0.93‐1.01)	.033	0.97 (0.93‐1.01)	.123
Low compliance	Duration of medication (M)						
	1‐12	118 (30.6)	4 (7.1)	1.00		1.00	
	13‐24	100 (26.0)	14 (25.0)	4.13 (1.32‐12.95)	.015	4.43 (1.4‐14.04)	.012
	25‐36	60 (15.6)	12 (21.4)	5.90 (1.83‐19.08)	.003	6.20 (1.89‐20.32)	.003
	37‐48	59 (15.3)	13 (23.2)	6.50 (2.03‐20.81)	.002	7.04 (2.17‐22.84)	.001
	≥49	48 (12.5)	13 (23.2)	7.99 (2.48‐25.74)	<.001	7.80 (2.39‐25.45)	.001
	Medication type						
	AIs	249 (64.7)	28 (50.0)	1.00		1.00	
	TAM	136 (35.3)	28 (50.0)	1.83 (1.04‐3.22)	.036	1.78 (0.99‐3.20)	.054
	No. of side effects						
	0	202 (52.5)	16 (28.6)	1.00		1.00	
	1	127 (33.0)	20 (35.7)	1.75 (0.88‐3.50)	.052	1.82 (0.90‐3.68)	.096
	≥2	55 (14.3)	20 (35.7)	4.04 (1.99‐8.21)	<.001	4.06 (1.96‐8.41)	<.001
	Unknown	1 (0.3)	0	—	—	—	—
	BMI	385 (100.0)	56 (100.0)	0.94 (0.87‐1.01)	.034	0.95 (0.88‐1.03)	.186

## 4 DISCUSSION

### 4.1 Adherence status to AET in Chinese women with early breast cancer

Despite the proven benefits of AET, patient persistence on and compliance to tamoxifen and AIs are suboptimal. Persistence with adjuvant endocrine therapy steadily declined over time in women receiving adjuvant endocrine therapy for early breast cancer in China. Women taking tamoxifen in the 2nd to 3rd year of medication and AIs in the 1st to 2nd year showed the greatest decrease.

The nonpersistence rate of 13.4% in our study was similar to that of Japan (12%).^20^ Our discontinuation rate of 22.6% at the fifth year is lower than that of most previous reports, which estimated discontinuation rates ranging from 31% to 73%.^11^, ^12^, ^21^, ^22^ Higher than an USA study of 538 women with 18% non‐persistent at 5 years,^23^ similar to the NSABP B‐14 trial, whose discontinuation rates were 23% in both the placebo group and the experimental group for 60 months.^24^ Our study population included patients from one university cancer hospital, where all women had continuous medical care; considering the different calculation methods of discontinuation rate, this study only calculated the patients who simply stopped using the drug, and did not calculate the switch drug replacement; all these factors may help explain our low discontinuation rate.

Compliance is defined as the consistency of taking protocol treatment; a patient was compliant if she took at least 80% of the pills during each month. The results of this study showed that 89.09% of the patients self‐reported taking more than 80% of their doctor's prescription pills each month. A systematic review identified 29 studies of adjuvant endocrine treatment adherence: compliance ranged from 41% to 72%.^13^ Several studies have suggested that information and support improve adherence, lending credence to this possibility.^25^, ^26^ The mean medication compliance score of breast cancer patients was 0.853, and 50.6% of patients had high compliance. It is similar to the research of Kesmodel et al which respectively showed 50% of 100 cases of early breast cancer with high compliance.^27^

### 4.2 Analysis of influencing factors associated with adherence

#### 4.2.1 Type of medication

The type of medication is another factor affecting adherence to endocrine therapy in this study. Women who used tamoxifen had significantly increased odds of nonpersistence compared with women who used AIs. As in several prior reports, women who discontinued endocrine therapy were more likely to have used tamoxifen compared with AIs.^12^, ^23^ Lack of awareness of tamoxifen is an important factor affecting compliance.^28^ Misunderstanding and fear of side effects of tamoxifen also affect medication compliance. A Los Angeles study also points to inadequate information about side effects as a factor in the persistence of medication.^29^ Other studies have shown that depression is associated with early discontinuation of tamoxifen.^30^ It is suggested that special attention should be paid to the patients taking tamoxifen in the future research and clinical practice, and effective methods should be adopted to ensure that the side effects of patients can be targeted intervention and the knowledge related to endocrine drugs can be timely acquired.

#### 4.2.2 Duration of medication

Analyses showed that the 2nd to 3rd year of endocrine therapy was the most apparent period of the increase of medication interruption rate and decrease of compliance rate in breast cancer patients. Fontein et al also found that patients with adjuvant endocrine therapy had a high interruption rate after 2.5 years of treatment.^31^ After the basic treatment, patients began to take endocrine therapy drugs, which were convenient to take and had fewer side effects than radiotherapy and chemotherapy. The patients were willing to stick to the medication rather than go back to the original process. As treatment continued, side effects began to appear. Some patients think they are in good physical condition, but their perception is more about the troubles and intolerable side effects brought by the treatment.^28^ Patients' awareness and attention to endocrine therapy decreased, their belief in taking medicine gradually decreased, and their fear of tumor recurrence and metastasis also decreased.^32^, ^33^At this point, their compliance with the medication becomes worse. This may explain why endocrine therapy produces the first peak of recurrence in 2‐3 years.

#### 4.2.3 Side effects

Among the 888 patients followed‐up, 442 (49.8%) reported side effects, and 139 patients had more than two side effects. Of the 119 patients who discontinued the medication, 55 (46.22%) reported side effects, and 30.25% discontinued the medication as a result of side effects. Of the 769 patients who took the medication consistently, 50.2% reported side effects, and 69.9% of patients with low compliance had at least one side effect. The rate of side effects in this study was similar to a New Zealand study in which 52.5% of patients had side effects and 30.9% of patients stopped taking drugs due to side effects.^22^ A survey of women in Detroit and Los Angeles who started endocrine therapy showed that of those who stopped early, 40% stopped for side effects and 25% cited worry about risks.^29^ Another Chinese study showed that 36% of patients discontinued endocrine therapy because of its side effects, another 14% stopped treatment after reading the package insert about side effects rather than after having experienced by themselves.^34^ The adverse effects may be unbearable, life threatening, or decrease quality of life.^28^ Side effects are the main factors affecting compliance to adjuvant endocrine therapy.^35^, ^36^, ^37^

#### 4.2.4 Other factors

The results of this study showed that older than 60 years and younger than 39 years patients were more likely to be non‐persistent, which was similar to the results of several studies.^13^, ^35^, ^38^, ^39^, ^40^ Younger women may be more likely to make a risk‐benefit assessment rejecting AET because AET is associated with side effects. In addition, tamoxifen is a SERMS drug, and young women may prioritize fertility issues, while older women may consider comorbidities, life expectancy, and quality of life.^28^, ^41^

A univariate analysis found that whether a patient had been treated with radiation had an effect on medication persistence. However, further multivariate analysis showed that radiotherapy history was not one of the factors influencing nonpersistence. Whether the history of radiotherapy is related to the early interruption of treatment is inconsistent with the existing studies.^20^, ^31^, ^34^ One possible explanation is that patients who did undergo radiotherapy may be more likely to receive standard‐of‐care therapy in general.^41^

The lack of caregivers during endocrine therapy affected the persistence of treatment, similar to the study results of Cluze et al 's follow‐up of 196 breast cancer patients taking tamoxifen, which found that treatment interruption was mainly due to lack of social support.^42^

In this study, univariate analysis showed that body mass index (BMI) as a continuous variable had an impact on compliance, but it was not significant when BMI was changed into ranked data for analysis. There have been several studies, which associated higher BMI with lower efficacy of antihormonal treatment.^43^ Being overweight or obese was a significant time dependent factor predictive for discontinuing endocrine therapy. But this was not found in our study, the reason may be that the BMI data we selected were at the time of diagnosis. As the treatment progresses, the patient's BMI may change, especially the weight gain caused by endocrine therapy; medication compliance will reduce. However, we did not collect the BMI data at that time. BMI influence on compliance needs further research.

#### 4.2.5 Study limitations

This study has several limitations. First, a total of 888 cases were included in this study, and only 146 people were in their fifth year of endocrine therapy, which is a relatively small sample size compared to the first 2 years. In future studies, the sample size can be further expanded, and the prospective follow‐up investigation on the samples will continue. Second, the nonpersistence rate of 13.4% in our study was lower than most previous studies.Considering the different calculation methods of interruption rate, our study only calculated the patients who stopped the drug, and the patients who changed the drug did not calculate the interruption rate. Future research should adopt unified evaluation of discontinuation rate calculation method to obtain comparable results. Third, there was a recall bias in our study, cross‐sectional follow‐up was used to understand the medication status of patients during the first 5 years of treatment in the same period to decrease bias caused by recall bias and environmental causes. Finally, we applied nine additional questions to help us understand the entire state of the patient's current treatment, to get a sense of possible factors that might affect treatment compliance, and since it was not a formal questionnaire, we did not test these questions for consistency and feasibility.

#### 4.2.6 Clinical implications

The results of this study found that the time of changes in nonpersistence rate and compliance rate in AET patients provided a reference for the selection of clinical intervention time. This study suggests that more attention and timely intervention should be given to patients receiving endocrine therapy before they show obvious nonpersistence, and clinicians should emphasize the importance of completing the 5‐year treatment to patients.

## 5 CONCLUSION

Medication adherence is affected by many factors. Special attention and interventions should be given to women taking tamoxifen for the 2nd to 3rd year of medication, and to women taking AIs for the 1st to 2nd year. Future research should adopt unified evaluation of discontinuation rate calculation method to obtain comparable results. Further prospectively designed studies are needed to explore the factors affecting medication adherence in patients undergoing endocrine therapy for breast cancer. 

## AUTHOR CONTRIBUTIONS

Hui Xu carried out data analysis, interpretation, and wrote the paper. Ai‐ping Wang provided guidance on study design, organized the investigation, and is the corresponding author. Feng Jin provided guidance on study design and interpretation of analysis. Xiu‐jie Zhang, Da‐qiu Wang and Shao‐fen Yu provided help with the data collection, analysis, and interpretation. All authors read and approved the final manuscript. The authors declare that there is no conflict of interest regarding the publication of this article.

## Data Availability

The data that support the findings of this study are available from the corresponding author upon reasonable request.
